# Endotracheal intubation results in acute tracheal damage induced by mtDNA/TLR9/NF‐κB activity

**DOI:** 10.1002/JLB.5A0718-254RR

**Published:** 2018-12-13

**Authors:** Carlos A. Puyo, Alexander Earhart, Nicholas Staten, Oliver A. Prince, Colleen Haug, Marin Kollef, Michael Awad

**Affiliations:** ^1^ Departments of Anesthesiology and Critical Care Washington University School of Medicine in Saint Louis St. Louis Missouri USA; ^2^ Internal Medicine Washington University School of Medicine in Saint Louis St. Louis Missouri USA; ^3^ Surgery Washington University School of Medicine in Saint Louis St. Louis Missouri USA

**Keywords:** endotracheal intubation, mitochondrial DNA, neutrophils, pain, TLRs

## Abstract

Tracheitis secondary to placement of an endotracheal tube (ETT) is characterized by neutrophil accumulation in the tracheal lumen, which is generally associated with epithelial damage. Mitochondrial DNA (mtDNA), has been implicated in systemic inflammation and organ dysfunction following trauma; however, less is known about the effects of a foreign body on local trauma and tissue damage. We hypothesized that tracheal damage secondary to the ETT will result in local release of mtDNA at sufficient levels to induce TLR9 and NF‐κB activation. In a swine model we compared the differences between uncoated, and chloroquine (CQ) and N‐acetylcysteine (NAC) coated ETTs as measured by tracheal lavage fluids (TLF) over a period of 6 h. The swine model allowed us to recreate human conditions. ETT presence was characterized by neutrophil activation, necrosis, and release of proinflammatory cytokines mediated by TLR9/NF‐κB induction. Amelioration of the tracheal damage was observed in the CQ and NAC coated ETT group as shown in tracheal tissue specimens and TLF. The role of TLR9/NF‐κB dependent activity was confirmed by HEK‐Blue hTLR9 reporter cell line analysis after coincubation with TLF specimens with predetermined concentrations of NAC or CQ alone or TLR9 inhibitory oligodeoxynucleotide (iODN). These findings indicate that therapeutic interventions aimed at preventing mtDNA/TLR9/NF‐κB activity may have benefits in prevention of acute tracheal damage.

## Graphical abstract

Interaction between a foreign body and the trachea, as mediated by the innate immune system.

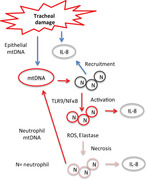

**Abbreviations**
7AAD: 7‐aminoactinomycin D
CQ: chloroquine
DAMP: damage associated molecular pattern
ETT: endotracheal tube
HEK: human embryonic kidney (cells)
iODN: inhibitory oligodeoxynucleotide
mtDNA: mitochondrial DNA
NAC: N‐acetylcysteine
ODN: oligodeoxynucleotide
PRR: pattern recognition receptor
TLF: tracheal lavage fluid

## 1 INTRODUCTION

Placement of an endotracheal tube (ETT) is an essential procedure performed during different aspects of medical care. The tracheal mucosa is exposed daily to a myriad of endogenous and exogenous agents that are recognized and controlled by the innate immune system, in particular, by neutrophils. Placement of an ETT is a procedure capable of inducing various degrees of tracheal tissue damage,^1^, ^2^, ^3^ pain,^4^ tracheitis, and possibly pneumonia.^5^ Although studies have shown the effects of airway pathogens on local and systemic inflammation,^6^ the early events that lead to tracheal neutrophil activation in the absence of infection have not been elucidated. Neutrophil luminal migration implies a barrier disruption of the epithelial layers that is suggestive of a neutrophil/epithelial interaction as is indicated by the surface protein expression of ICAM‐1 (CD54).^7^ Activated neutrophils have been implicated in disruption of cellular viability^8^, ^9^, ^10^, ^11^ that is mediated by multiple cytokines such as TNF‐α,^12^ IL‐1β,^13^ and IL‐6.^14^ Indeed, we have shown previously that contact between the ETT and the human or swine tracheal mucosa results in mechanically induced injury characterized by sore throat (humans), tracheitis, and induction of an activated neutrophil phenotype^15^, ^16^, ^17^ as well as simultaneous elevation of elastase, reactive oxygen species (ROS), IL‐1β, TNF‐α, and expression of ICAM‐1.^17^

The innate immune system and, in particular, neutrophils are necessary for an organized response against pathogens and host generated danger signals. Neutrophils possess conserved pattern recognition receptors (PRR),^18^, ^19^ among them TLRs^20^ capable of recognizing antigens originated during sterile and nonsterile cellular injury. Notably, injury induces release of host cellular molecules known as damage associated molecular patterns (DAMPs)^21^ that are readily recognized by TLRs. It is known, that the respiratory system expresses multiple TLRs including TLR2, TLR4, and TLR9 on cells lining the airway.^22^, ^23^ TLR9 activation through recognition of bacterial unmethylated CpG DNA^24^ or by oxidized mitochondrial DNA (mtDNA)^25^, ^26^, ^27^ of nonbacterial origin are examples of innate recognition of nonself and self‐antigens, respectively, during injury. Recent studies have demonstrated that oxidized mtDNA released during eukaryotic cell injury^25^, ^26^, ^27^, ^28^ is a danger signal that may induce a potent immune mediated inflammatory response following engagement of TLR9. Organ dysfunction secondary to mtDNA release and induction of sterile injury has been demonstrated during cardiac dysfunction,^28^ and tracheal^17^ and lung injury.^25^ Previously, in human subjects who received an ETT we documented higher concentrations of mtDNA and TLR9 in aseptic tracheal lavage fluids (TLF) of patients suffering from sore throat as compared to those without sore throat.^17^ Signaling of TLR9 is known to occur via MyD88 resulting in transcription of several hundred genes mediated by NF‐κB.^29^, ^30^ Activation of NF‐κB has been observed in studies of systemic inflammatory response syndrome (SIRS),^31^ bowel inflammation,^32^ and arthritis,^33^ as well as in our human tracheal study,^17^ resulting in gene expression of proinflammatory cytokines.

There is no effective method to ameliorate tracheal damage induced by mtDNA release secondary to a foreign body. In this study, we hypothesize that tracheal tissue damage induced by an uncoated ETT would promote neutrophil activation, migration, and necrosis via TLR9/NF‐κB activation. We additionally aimed to determine the effects of medications chloroquine (CQ; anti‐inflammatory) and N‐acetylcysteine (NAC; ROS scavenger) as a coating for ETT tubes to limit cellular injury.

## 2 MATERIALS AND METHODS

### 2.1 Study design

After approval by the Animal Studies Committee at Washington University in St. Louis, and in accordance to the guidance stipulated by the Animal Welfare Act and the Association for the Assessment and Accreditation of Laboratory Animal Care (AAALAC), we conducted a prospective study in swine to evaluate the impact of an ETT on tracheal tissue and neutrophil activation during 6 h of exposure. We randomized the swine to two groups (5 animals each), one uncoated and the other CQ/NAC coated. We utilized a 7 mm ID Mallinckrodt™ TaperGuard Evac ETT that was dipped in a mixture of CQ/NAC in 20% Polyvinylpyrrolidone (PVP)/10% ethanol for 15 min and dried in a sterile incubator at 37°C +5% CO2. This coating and drying cycle was repeated two more times before the coated devices were returned to their original sterile packaging. Identical ETTs without coating were utilized in the other group of animals.

Ten healthy female pigs were anesthetized with 1 to 2 mg/kg of tiletamine, ketamine, xylazine (TKX) prior to intubation, and anesthesia was maintained with 1–3% isoflurane. ETT balloons were inflated with 10 mL of air titrated to cuff leaks at 20 cm/H_2_O pressure. Tracheal lavages were done at 0 (5‐10 min after intubation), 3 and 6 h, with 10 ml of sterile saline solution using a push and suction technique with a wall mounted device set up at low suction pressures. Blood was also drawn from a peripheral vein at 0, 3, and 6 h. After ETT removal, pigs were euthanized and biopsies of the trachea at the point of contact were collected and preserved in formalin.

### 2.2 Neutrophil phenotypes and respiratory burst assessment

Cell pellets from the TLF specimens after centrifugation were manually broken apart by pipetting, and neutrophils were isolated by magnetic negative separation using EasySep™ Neutrophil Enrichment Kits (Stem Cell Technologies, Tukwila, WA, U.S.) (Fig. 2A). Isolated neutrophils were stained with fluorochrome‐conjugated antibodies for CD11a, CD11b, CD16, CD18, CD54, and CD62L for assessing activity and adhesion (Fig. 2C). 7‐Aminoactinomycin D was used to assess cellular necrosis and Annexin V as a viability marker (Fig. 2B). Neutrophil respiratory burst was characterized by incubating cells for 10 min at 37°C with 10 ng/mL phorbol 12‐myristate 13‐acetate followed by 10 s with 20 μM dihydrorhodamine 123 (DHR123) (Fig. 6A). Neutrophil phenotypes and ROS were characterized by FACS and analyzed with FlowJo^®^ X software (FlowJo, LLC). Total RNA was isolated from the TLF neutrophils from both groups via TRIzol^®^ (Thermo Fisher Scientific, Carlsbad, CA, U.S.).

### 2.3 Mitochondria DNA quantification

TLF obtained from each time point were centrifuged, and supernatants were collected to quantitate mtDNA by quantitative polymerase chain reaction (qPCR) for swine cytochrome b (Mt‐cyb) compared against a known concentration of mtDNA (Fig. 3). RT‐PCR using primer/probe pairs specific to porcine cytochrome b (*Mt‐cyb*), which is encoded in mtDNA but not encoded in genomic DNA made a distinction between mtDNA and total DNA. We compared the *Mt‐cyb* values to known concentration of *Mt‐cyb* DNA template provided by the primer manufacturer (qSscCEP 0027819, catalog numbers for primer 12001961, and template 10047280, Bio‐Rad Laboratories, Inc., Hercules, CA, U.S.).

### 2.4 TLR9 expression in TLF neutrophils

Comparative analysis for TLR9 expression of TLF neutrophils (Fig. 3B) was done using mRNA from blood neutrophils as a baseline control instead of using a separate reference gene. Quantification was done using a qPCR cycle threshold (Ct) method of relative quantification in which concentrations of mRNA from all samples were adjusted in reference to each other as evaluated with a nanodrop 2000, and as described elsewhere.^34^ We used Taqman TLR9 primer from Thermo Fisher Scientific with KAPA Probe Fast Master Mix.

### 2.5 HEK 293 TLR9 reporter assay

An HEK 293 reporter cell line (Fig. 4) transfected to produce excess TLR9 and NF‐κB (HEK‐293 Blue hTLR9; InvivoGen, San Diego, CA, USA) which induces secreted embryonic alkaline phosphatase (SEAP) production when activated, was grown in accordance with manufacturer instructions at 37°C +5% CO_2_, with passaging every 2 or 3 d. Following passaging 3 times, approximately 1 × 10^5^ cells in 100 μL growth media (made up of DMEM with 4.5 g glucose, 2 mM glutamine, 10% FBS), 20 U/mL penicillin/streptomycin, 100 μg/mL Normocin/Zeocin antibiotics) were plated for 30 min in 96‐well plates and then treated with 100 μL TLF from both uncoated and CQ/NAC‐coated swine groups for 4 h at 37°C +5% CO_2_, with 10 μM stimulatory oligodeoxynucleotide (ODN) ODN 2006 and inhibitory ODN TTAGGG A151 (inhibitory oligodeoxynucleotide [iODN]; InvivoGen) as both positive and negative controls. Negative controls were obtained by exposing HEK293 cell line to only the treatment drugs and with no exposure to ODN or TLF. TLR9 activation of NF‐κB was measured by calorimetric detection of SEAP production by the enzymatic hydrolysis of 100 μL QUANTI‐Blue detection substrate (InvivoGen) after 1 h of incubation at 37°C +5% CO_2_. This was measured with the Synergy H1 micro‐plate reader (BioTek Instruments, Inc., Winooski, VT, USA) at 655 nm absorbance.

### 2.6 Inflammatory markers in TLF

Extracellular concentrations of the inflammatory markers IL‐1β, IL‐6, IL‐8, IL‐10, and TNF‐α were determined by sandwich ELISAs (Fig. 5) against standard curves for each provided by the manufacturer (Thermo Fisher Scientific) for secretion. Enzymatic activity of neutrophil elastase in TLF (Fig. 6B) was measured by the breakdown of the chromogenic substrate N‐methoxysuccinyl‐Ala‐Ala‐Pro‐Val p‐nitroanilide (Sigma Aldrich) into 4‐nitroaniline and measured by spectrophotometric absorbance at 405 nm using a NanoDrop 2000 (Thermo Fisher Scientific).

### 2.7 Tracheal histology

Tracheal tissue biopsies at the point of contact with the ETTs were taken from both uncoated and CQ/NAC coated swine groups and fixed in 10% formaldehyde for 48 h, washed and dehydrated in ethanol, and paraffin‐embedded. Samples were then cut and stained with H&E. A veterinary pathologist blinded to the research conditions scored the histological specimens of at least 4 subjects for evidence of epithelial tissue injury/integrity, inflammation, and cellularity using a numerical scoring guideline scaled from 0 to 4, with 0 corresponding to normal or minimally injured tissue and no inflammatory cells, whereas 4 represented extensive tissue damage and elevated inflammatory cell infiltration (Table 1). Typically a swine trachea unexposed to an ETT has low cellularity (Supplemental Table 1).

**Table 1:** Comparative tracheal tissue histology

Variable	Uncoated	Coated	P
Criterion			
Fraction of basement membrane covered by epithelium	2.25	1.75	0.139
Mucosal pathology	3.75	3.37^b^	**0.009**
Submucosal pathology	2.75	2.5	0.296
Inflammation—neutrophils	2.37	2^b^	**0.008**
Inflammation—mononuclear cells	2.12	2.62^b^	**0.001**
Average total score	13.25	12.25^a^	**0.028**

Histology of swine tracheal tissues exposed to uncoated and CQ/NAC coated ETT. Scoring table for inflammatory cells represented by neutrophils, mononuclear cells and fraction of basal membrane that is covered by epithelial tissue. Lower scores represent better tissue preservation and higher scores correspond to tissue damage of various degrees. Tissues scored for pathology and infiltration of inflammatory cells.

a *P* < 0.05.

b *P* < 0.01 by Student's *t* test, considered significant.

### 2.8 Statistics

Concentrations of mtDNA were compared with Kruskal‐Wallis and post hoc Dunn's tests, whereas mean fluorescence intensity (MFI) from FACS analysis, ELISA, and NE activity data were compared against inactive peripheral blood neutrophils with Wilcoxon Mann‐Whitney *U* tests. All histology data was compared by a Student's *t* test. All data were analyzed using SPSS v.17.0 (SPSS, Inc., Chicago, IL, USA).

## 3 RESULTS

### 3.1 Tracheal epithelium is disrupted by uncoated ETT

**Figure 1:**
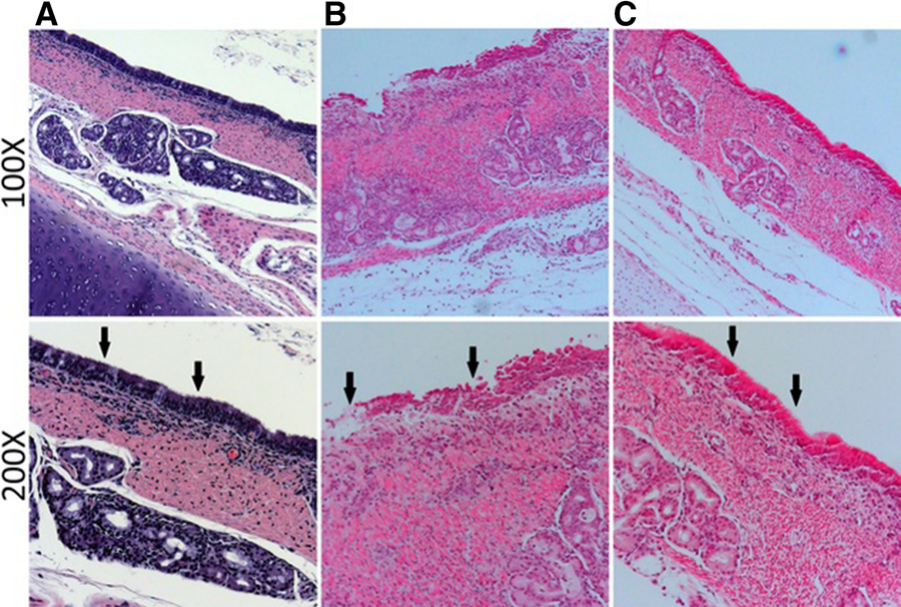
**Comparative Tracheal histology analyzed at the point of contact between the endotracheal tube and tracheal tissue**. Tissues representative of tracheal tissues exposed to direct contact with uncoated and CQ/NAC coated endotracheal tubes (ETTs). **1A)** Trachea from a normal swine with no ETT at 100× and 200× with arrows pointing to intact epithelium and ciliation. **1B)** Epithelial disruption with multicellular migration and magnified at 100× and 200× with arrows to highlight the extensive lack of ciliation and tissue damage in the areas of contact with an uncoated ETT. **1C)** Better preserved tracheal tissue with significant reduction of cellular infiltrate and magnified at 200× highlights presence of an intact epithelium and ciliated structures with arrows following exposure to a CQ/NAC coated ETT

Previously, we have shown that surrogate markers of tissue injury were present in TLF obtained from human and swine subjects exposed to an uncoated ETT.^17^ In the present study we aimed to analyze the impact of ETT exposure at 6 h. We compared the effects of an uncoated ETT against a CQ/NAC coated ETT, utilizing collected TLF samples and specimens from local tissues. Here we demonstrate in the H&E sections that in contrast to the normal tracheal tissue (Fig. 1A), placement of an ETT results in tracheal damage at the point of contact (Fig. 1B control). In contrast, tracheal tissue exposed to CQ/NAC coated ETT showed less tracheal epithelium damage (Fig. 1C; arrows). Furthermore, in Table 1 analysis of the histopathology shows that tracheal tissues with inflammation and higher cellularity in the uncoated ETT group (13.25), whereas the CQ/NAC coated group was significantly decreased (12.25, *P < 0.05). Tracheal tissue neutrophils were significantly decreased in the coated group (**P < 0.01). It is noteworthy that normal tracheal tissues have low cellularity prior to ETT exposure (Fig. 1A and Supplemental Table 2). However, following ETT exposure, epithelial disruption, and high cellularity are evident as indicated in Fig. 1B and Supplemental Fig. 1.

### 3.2 Luminal neutrophils and phenotypes associated with ETT induced damage

**Figure 2:**
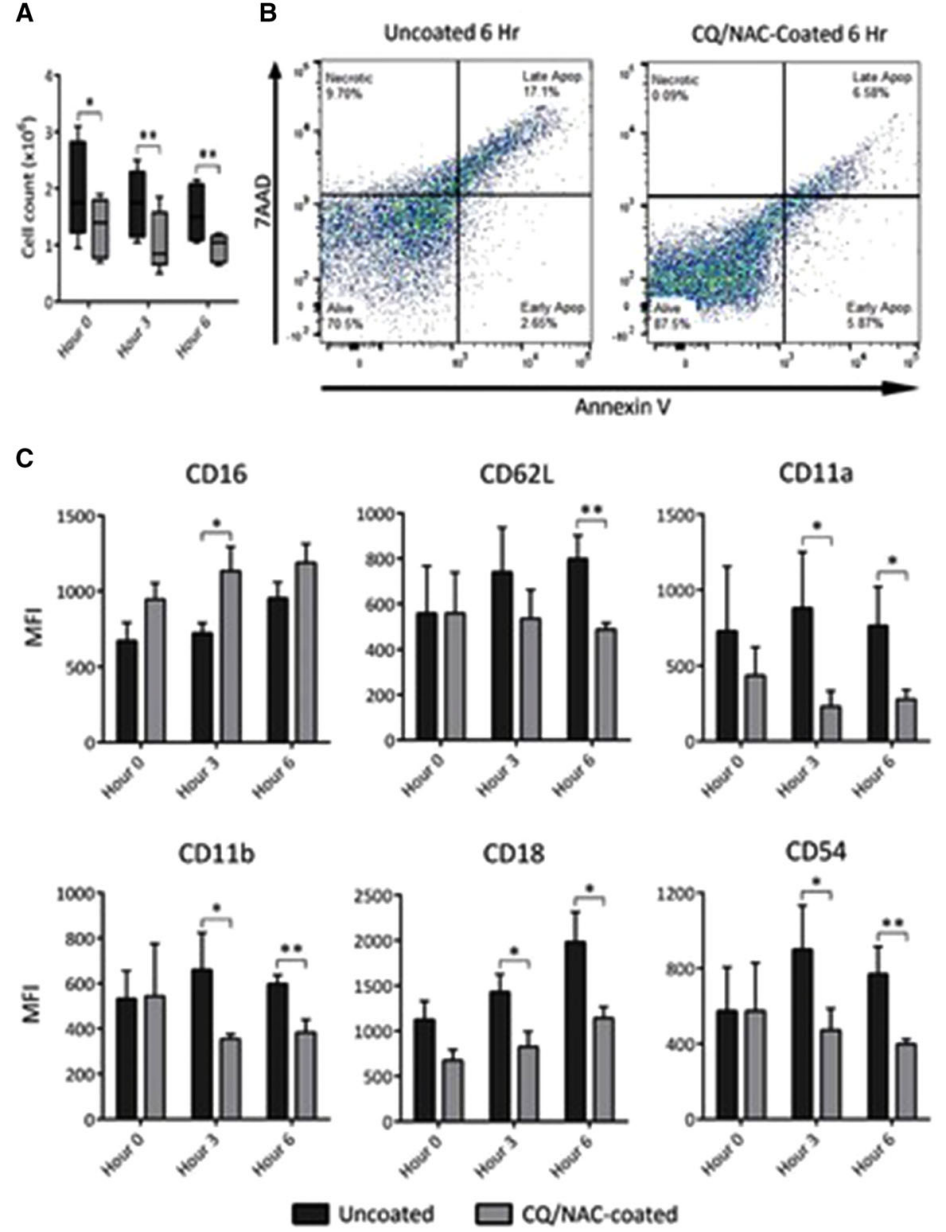
**Contact of swine trachea with an ETT promotes neutrophil migration, necrosis, and an activated neutrophil phenotype during presence of mtDNA**. **2A**) Total live neutrophils cell count was done by tryptan blue exclusion following negative immunomagnetic isolation in coated (*n* = 5, gray) and uncoated ETT (*n* = 5, black) groups over a period of 6 h. A high neutrophil count was observed in the uncoated group. **2B)** Cellular viability analysis was conducted in SSC^lo‐hi^ for AnnexinV^lo‐hi^ and FSC for 7AAD^lo‐hi^. Left FACS panel shows elevated necrosis (9.70%) and low early apoptosis (2.65%), high late apoptosis (17.2%) with live cells accounting for (70.5%) in the untreated ETT group, whereas, the right panel shows lower necrosis and late apoptosis and higher concentration of live cells in the coated group. Statistical analysis is reported in the supplemental data Fig. 2B. **2C)** Tracheal lavage fluid (TLF) analysis was conducted by flow cytometry in coated (gray bars *n* = 5) and uncoated ETT (black bars *n* = 5), with neutrophil cells identified by CD16^lo‐hi^/CD62L^lo‐hi^ monoclonal antibodies. Neutrophils with the phenotype (CD16^lo^/CD62L^hi^/CD11a^hi^/CD11b^hi^/CD18^hi^/CD54^hi^) are indicative of activation and migration as determined in TLF. Neutrophils of uncoated samples showed a higher surface expression of ICAM‐1 and integrins as compared to the uncoated group. Data shown represents MFI ± sem *P < 0.05; **P < 0.01, derived from 10 swine experiments

Acute innate immune response in a variety of tissues is mediated predominantly by neutrophils.^17^, ^35^, ^36^, ^37^ In order to understand the neutrophil response to the ETT, we analyzed TLF from subjects exposed to an ETT, and compared them to CQ/NAC coated ETT subjects. The uncoated ETT group was the control group because it reflects the current clinical standard of care. Therefore, we focus on the tracheal luminal neutrophils following migration after exposure to the ETT, and as indicated by Fig. 2A, we observed an elevation in the total neutrophil counts between coated and uncoated groups. This is in agreement with our observation in Fig. 1B, where we documented accumulation of inflammatory cells from tissues adjacent to the epithelial injury. Subsequently, we further analyzed the balance between necrosis and apoptosis, because the imbalance of these factors may have a role in initiation and perpetuation of tissue injury. We utilized cell viability staining on isolated neutrophils from the TLF obtained in both groups, and observed a statistically significant increase in necrotic cell staining in the uncoated ETT group (Supplemental Figs. 2A, B). In contrast, a decrease in markers of necrosis and increase in apoptosis was detected in the coated group (Fig. 2B, Supplemental Fig. 2); together these data show that persistent neutrophilia is a characteristic of ETT damage. Subsequently, we characterized neutrophil surface markers by FACS analysis of TLF neutrophils at 6 h by gating at SSC^hi^ CD62L^lo‐hi^ CD16^lo‐hi^. At 6 h, activated neutrophils showed surface expression of CD62L^hi^/CD11a^hi^/CD11b^hi^/CD18^hi^/CD54^hi^ corresponding to the uncoated ETT group (Fig. 2C, dark bars), whereas, the CQ/NAC coated group had a less activated phenotype (Fig. 2C, gray bars). Moreover, neutrophils from the uncoated ETT group showed higher surface expression consistent with adhesion and migration as determined by CD11a (LFA‐1), CD11b (Mac‐1) and the common β‐2 subunit (CD18).

### 3.3 Mitochondrial DNA/TLR9/NF‐κB pathway activation during injury

**Figure 3:**
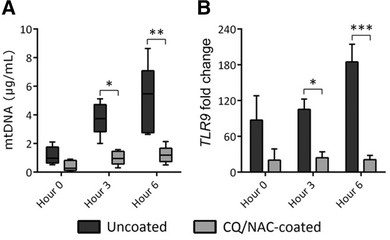
**Uncoated ETTs promote mitochondrial DNA (mtDNA) concentration and TLR9 expression**. Tracheal lavage fluid (TLF) specimens obtained from uncoated or CQ/NAC coated ETT over a period of 6 h were analyzed for: **3A)** mtDNA concentrations as determined by real time PCR using swine Mt‐cyb primer and a standard provided by the manufacturer, and shows higher concentration in the uncoated ETT group. **3B**) Fold change in TLR9 transcription in tracheal neutrophils from uncoated (black, *n* = 5) and coated (gray, *n* = 5) ETT groups were compared against blood neutrophils as base line, and determined by real time PCR against peripheral blood neutrophils. *P < 0.05, ***P < 0.001

**Figure 4:**
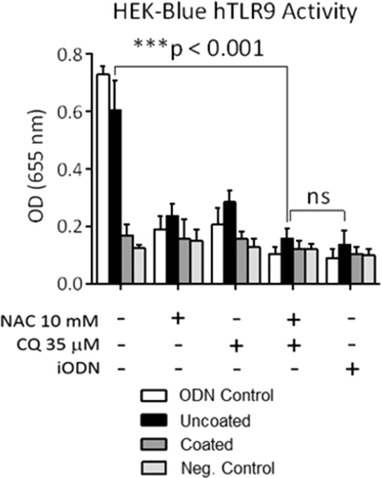
**Uncoated ETTs stimulate TLR9 signaling and NF‐κB activation**. Tracheal lavage fluids (TLF) of uncoated and CQ/NAC coated ETTs were co‐incubated with HEK‐Blue™ hTLR9 reporter cell line or pre‐incubated with graded concentrations of CQ/NAC, NAC or CQ alone or TLR9 iODN. Negative controls represent exposure of the cells line to treatments but no stimulus with ODN or TLF. A human embryonic kidney 293 (HEK 293) cell line containing TLR9 and NF‐κB/secreted embryonic alkaline phosphatase (SEAP) genes (HEK‐Blue™ hTLR9) was use to analyzed the supernatant for SEAP activity under control of transgene of NF‐κB/AP‐1 by measuring absorbance at 655 nm after 6 h of incubation. Data is representative of triplicate experiments where mean activity + SD was calculated from 5 coated and 5 uncoated TLF specimens and considered significant if ***P < 0.001. HEK—human embryonic kidney; iODN—inhibitory oligodeoxynucleotide; NAC—N‐acetylcysteine; CQ—chloroquine

The release of mtDNA is a byproduct of cellular damage that is well established; thus we aimed to quantify mtDNA in the TLF in our study (6 h). As indicated in Fig. 3A we were able to detect under 2 μg of mtDNA from both study groups. Consistent with acute injury, we detected statistically significant high amounts of mtDNA in TLF of the uncoated specimens at 6 h, which contrast the observed levels in the CQ/NAC coated group (Fig. 3A). These data are consistent with our hypothesis comparing coated and uncoated ETT at 6 h. Subsequently, we used TLR9, a known receptor for mtDNA, as an indirect marker to assess the effect of increased mtDNA (Fig. 3B). As expected, we detected increased *TLR9* transcript in the TLF from the uncoated group consistent with the increased mtDNA in this group (Fig. 3). To further assess the impact of mtDNA found in TLF and the concomitant stimulation of TLR9, we sought to validate our results using an in vitro model based on the HEK293 TLR9/NF‐κB cell reporter line (Fig. 4). After exposure of the cell line to the TLF obtained from coated and uncoated subjects, we observed TLR9/NF‐κB activation in the uncoated samples (OD 0.6), similar to the positive control, ODN (OD 0.7). Collectively, these data support the role of the mtDNA/TLR9/NF‐κB pathway in acute ETT induced tracheal injury.

### 3.4 Increased IL‐8 is associated with acute injury in uncoated ETT

**Figure 5:**
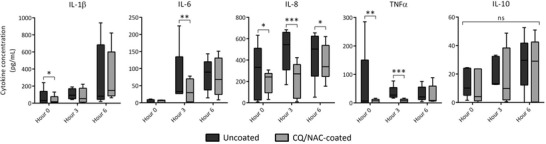
**Treated ETTs ameliorate luminal neutrophil secretion of proinflammatory cytokines**. Tracheal luminal neutrophils of CQ/NAC coated and uncoated specimens were exposed to predetermined amounts of chloroquine and N‐acetylcysteine and assessed for secretion by quantitative sandwich ELISA, respectively, for cytokines IL1β, IL‐6, IL‐8, TNF‐α, and IL10. ELISA of TLF neutrophil analysis showing secreted cytokines with a favorable response to treatment with CQ/NAC, noted in the gray bars. Data represents 5 independent experiments per group where mean levels ± sem *P < 0.05; **P < 0.01, ***P < 0.001 were considered significant

Because neutrophilia is a hallmark of tracheal epithelial damage, we investigated the presence of cytokines in the TLF with particular emphasis in the chemokine IL‐8. We confirmed secretion of proinflammatory cytokines IL‐1β, TNF‐α, IL‐6, IL‐8, and the anti‐inflammatory IL‐10 (Fig. 5) in the TLFs. We recognize that collection of TLF might have introduced some dilution in the protein readouts, which may explain some of the variability. Despite this observation, IL‐8, a known potent chemokine for neutrophils, was consistently elevated as at the 6 h time point for the uncoated group (Fig. 5). Taken together, therapeutic coating of ETT tubes results in significant secretion of anti‐inflammatory IL‐10 and a corresponding decrease in proinflammatory cytokine IL‐8, a potent neutrophilic chemokine.

### 3.5 Impact of coated ETT on ROS and elastase activity

**Figure 6:**
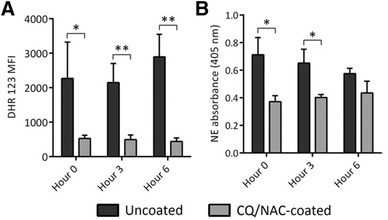
**Uncoated ETT promotes neutrophil reactive oxygen species (ROS) and elastase activity**. **6A**) Tracheal lavage fluids (TLF) of uncoated ETT (dark bars) shows neutrophil high mean fluorescence of ROS after co‐incubating phorbol 12‐myristate 13‐acetate with the dye staining dihydrorhodamine 123 (DHR123) and analyzed by flow cytometry. TLF of CQ/NAC coated ETT (gray bars) shows less ROS activity over the 6 h of the experiment. **6B**) Tracheal neutrophil elastase activity analyzed over a period of 6 h for the coated and uncoated ETT groups was assayed by hydrolysis of the chromogenic substrate N‐methoxysuccinyl‐Ala‐Ala‐Pro‐Val p‐nitroanilide (Sigma Aldrich, Inc., St. Louis, MO, USA) by light absorbance at 405 nm after 1 h incubation and analyzed using spectrophotometry. Neutrophil elastase activity was persistently higher in the uncoated ETT group (dark bars) in contrast to the treated ETTs (gray bars). Data shown represents results form 10 swine experiments were mean levels ± sem were considered significant if *P < 0.05; **P < 0.01

Excessive levels of ROS and elastase have significant impact on cellular activity and tissue damage. As indicated in Fig. 2A, the numbers of neutrophils in the TLF was persistently elevated in the uncoated group, and thus the damage induced by intubation is associated with ROS and elastase production (Fig. 6A, B). The apparent reduction of ROS in the coated group likely relates to the scavenging capacity of NAC, while the decrease neutrophil count likely corresponds to a lower cytokine activity mediated by CQ or possibly a combination of both medications (Fig. 6A, B). Neutrophil elastase showed no statistical differences at 6 h. These data supports the effectiveness of the ETT coating in ameliorating overall tracheal tissue damage by decreasing neutrophil accumulation, and scavenging ROS (NAC).

## 4 DISCUSSION

We present a model of tracheal luminal damage whereby placement and duration of an ETT exposure results in acute epithelial damage, neutrophilia, and DAMP release. The acute damage is characterized by localized release of mtDNA from epithelium and activated neutrophils. Activated neutrophil responses to mtDNA promote ROS and elastase secretion as well as necrosis. As a result of mtDNA release, a rapid activation of TLR9/NF‐κB results in potent IL‐8 production and accumulation of local neutrophils. We further postulate that prolonged exposure to an ETT would result in uncontrolled neutrophilic accumulation and ROS activity, thus promoting opportunistic bacterial colonization and biofilm formation. Studies have shown evidence of tracheal inflammation, edema, ulceration, granuloma, and fibrotic tissue formation that is promoted by indwelling ETTs.^1^, ^2^, ^3^, ^4^ The tracheal tissue is constantly exposed to antigens from external and internal sources and relies in an efficient mobilization of the innate immune system in particular neutrophil cells to provide a timely response.^35^, ^36^, ^37^, ^38^ Placement of an ETT results in trauma and provokes an exuberant response from the innate immune system increasing susceptibility to pain,^17^, ^39^ infection,^40^ and tissue damage.^17^, ^41^ The end result of this activation is variable, ranging from sore throat (humans) to tracheitis^15^, ^16^, ^17^, ^40^ and tracheal stenosis.^42^ Our study provides evidence of histological features representing tissue damage associated with the ETT, heralded by neutrophil luminal migration, extravasation, and accumulation in tracheal tissues. In contrast, tracheal tissue histology following exposure to the anti‐inflammatory benefits of CQ and the anti‐oxidant activity of NAC showed preservation of the epithelium and ciliary structures of the trachea. Neutrophil migration and extravasation is a characteristic of several inflammatory diseases,^43^, ^44^ and as we demonstrated here it is also present in ETT mediated tracheal damage. We have previously documented presence of tracheal neutrophilia in human and swine during ETT exposure,^15^, ^16^, ^17^ and in the present study we searched for evidence of functional changes consistent with neutrophil cell activation.

Different neutrophil phenotypes have been described in circulating neutrophils during acute systemic inflammation.^45^, ^46^, ^47^, ^48^ Here, we report that local TLF neutrophils obtained from the uncoated ETT group showed an activated neutrophil phenotype as determine by higher mean membrane expression of the adhesion molecules CD62L, CD11a, CD11b, CD18, and CD54, whereas lower expression was observed in the coated group. Interestingly, elevated mean neutrophil adhesion molecule expression has been documented in a variety of medical conditions including those of autoimmune origin. For example, in patients with systemic lupus erythematosus an elevated expression of the L‐selectin (CD62L), adhesion molecule has been reported in the serum of patients with active disease,^43^, ^44^ whereas, elevated expression of the β2‐integrins (CD11b/CD18) and CD54 have been documented in neutrophils during infection or ischemia‐reperfusion mediated tissue injured.^47^, ^48^ An elevated CD11b expression may have a prominent role in neutrophil recruitment during tracheal tissue injury, as it is required for transmembrane migration.^17^, ^49^, ^50^ The expression of certain adhesion molecules promotes further communication among neutrophils as noticed during CD54 (I‐CAM1) binding to CD11b, and with other CD11b expressing myeloid cells such as macrophages.^50^, ^51^

Although the life cycle of a neutrophil is short in the circulation, following migration and activation, apoptosis can be delayed as they migrate and enter intraluminal spaces.^38^, ^50^ Necrosis is a process known to result in cell lysis after exposure to highly toxic substances that culminate in release of DAMPs, among them mtDNA which result in induction of an immune‐inflammatory process.^52^, ^53^, ^54^ Here, we demonstrated that luminal neutrophil cells not only accumulate in the tracheal lumen but also undergo significant necrosis and likely represent a significant source of mtDNA during tracheal injury. Although we did not attempt to directly block TLR9 in vivo, we observed that exposure to an ETT results in necrotic bias of neutrophils under conditions of elevated mtDNA/TLR9 presence (Fig. 2A,B). As a result of cellular necrosis several local changes may result in tissue damage including release of mtDNA, which in turn activates TLR9 and may promote further release of ROS and tissue damage. ROS is essential for multiple homeostatic cellular functions, but uncontrolled activity results in cell injury^55^, ^56^, ^57^ during infections, or sterile injury by triggering NET formation,^57^ induction of NF‐κB transcription and possibly autophagy. Although ROS activity and lysosome acidification are important for TLR9 signaling in macrophages, the impact of these factors in neutrophils is less certain. Our data suggest that the same factors may be necessary for neutrophil TLR9 signaling as observed by the elevation in ROS and TLR9 transcription in neutrophils obtained from uncoated TLF (Figs. 3B, 6A). Interestingly, cell surface expression of TLR9 has been reported in neutrophils as a possible alternative to endosomal activation following exposure to pathogen ligands.^58^

Certainly, TLR9 presence as demonstrated in the current study may be a factor in delaying apoptosis,^38^, ^59^, ^60^ and exacerbating local inflammation; thus it is important to control TLR9 activation in order to preserve cellular homeostasis. Delayed neutrophil apoptosis may promote release of intracellular granules such as azurophilic granules, which contain harmful mediators such as elastase, and cathepsin G, both involved in pain induction.^61^, ^62^ For instance, neutrophil elastase has been shown to promote necrosis, mucus secretions, denudation of the respiratory epithelium, and decrease ciliary activity, as well as induce stimulation of IL‐8, a chemokine important in neutrophil migration.^48^, ^63^ Another important role of elastase in tissue injury relates to its presence in secreted neutrophil extracellular traps (NETs) in response to bacteria or tissue damage.^41^, ^64^, ^65^ Therefore, interest in ameliorating elastase activity has been the focus of several therapeutic interventions and as illustrated in our study, decreased elastase release appears to correlate with less tissue damage. Interestingly, elastase secretion combined with gene expression and secretion of IL‐1β and TNF‐α as observed in our study may have implications in induction and amplification of pain, as documented in other studies.^66^, ^67^ The importance of neutrophil elastase is highlighted by its presence in human and swine TLF, where it most likely exacerbates cell migration and tissue damage after neutrophil cell expression of CD11b/CD18/ICAM‐1. Expression of adhesion molecules and elastase activity was noticeably decreased in the CQ/NAC coated ETT group.

TLR9 activation triggered either by recognition of CpG of bacterial or nonbacterial origin initiates a signaling cascade with a vast impact on inflammation in particular via NF‐κB activation,^25^, ^68^, ^69^ partly promoting neutrophil trafficking, phagocytosis, and cytokine release.^70^, ^71^ Cell surface and lysosomal activation of TLR9 has been reported previously.^61^, ^72^, ^73^ Here we can only speculate that CQ decreased TLR9 activation induced by ETT damage, but the precise site of action remains to be elucidated. We aimed at neutralizing TLR9 activation with the use of the anti‐inflammatory agent CQ,^72^, ^73^ and sought to interfere with NF‐κB activation with NAC.^74^, ^75^ We investigated if TLR9 activation was inducing inflammatory cytokine gene expression in the uncoated ETT group. We approached this question using two different paths. Initially, we used an HEK293 reporter cell line to detect TLR9 signaling, and demonstrated that uncoated TLF was sufficient to drive NF‐κB and AP‐1 dependent gene expression. Coincubation of swine TLF from uncoated ETTs with a reporter cell line HEK293 indicated that mtDNA stimulates TLR9 activation. The uncoated ETT group had significantly more TLR9 activity and NF‐κB gene expression, than the coated ETT group. Subsequent use of CQ/NAC and iODN for treatment of uncoated TLF specimens also prevented TLR9 signaling, thus serving as confirmation of our initial observations. Moreover, this intervention not only resulted in inhibition of tissue damage mediated by an uncoated ETT but also in less ROS and elastase activity. Ligation of the TLR receptors and in particular the TLR9 receptor has been shown to exert broad impact on neutrophil activation,^17^, ^76^ effects also demonstrated in our study.

Although cytokine transcription and secretion are essential for tissue homeostasis, uncontrolled activity may exacerbate tissue damage by imbalance of cell and humoral immunity. Release of inflammatory mediators such as cytokines and chemokines takes place immediately after trauma. For instance, elevated concentrations of IL‐1β, IL‐6, IL‐8, and TNF‐α were documented in a study of patients with SIRS, whereas IL‐6 and IL‐10 were correlated with poor prognosis.^77^ Interestingly, IL‐10 presence has been documented in autoimmune disease processes in which a deficiency may result in intestinal inflammation.^78^ IL‐1β is also implicated in inflammasome activity in a caspase‐1 dependent manner illustrating the complexity of the local tissue homeostasis. Similarly, we observed that neutrophils obtained from TLF of swine receiving uncoated ETTs had a significant level of proinflammatory cytokine secretion. Up‐regulation of NF‐κB mediated cytokine gene expression was also demonstrated in our study, an effect that was ameliorated after the use of predetermined amounts of CQ/NAC in the coated ETTs.

Some outstanding questions remain regarding neutrophil activation mediated by aseptic molecules. We need to consider potential cytosolic mtDNA inflammatory targets such as inflammasomes^79^ and the cGAS/STING pathways in future studies. Studies have indicated that cytosolic activity of mtDNA has an important role in inflammasome activation by cleavage of caspase‐1 and maturation of IL‐1β and IL‐18. Interestingly, we detected IL‐1β in TLF during tracheal damage, suggesting a possible role in the nucleotide‐binding domain and leucine‐rich repeat (LRR)‐containing (NLR) family and the pyrin and HIN domain (PYHIN) NLRP3 family. The importance of the NLRP3 inflammasome in immunity and human diseases has been well documented, but the mechanism and regulation of its activation remains unclear. Another group of cytosolic proteins with relevance for future studies include the cyclic GMP‐AMP (cGAMP) synthetase (cGAS), and the stimulator of interferon genes protein (STING) pathways, which participate in innate immune responses mediated by mtDNA activity, and are important in the completion of the apoptotic cellular process mediated by caspases 3, 7, and 9.^80^

Although neutrophils have the capacity to release mtDNA following cellular necrosis, other cells such as macrophages, and epithelial cells may also release DAMPs (HMGB1); therefore they need to be evaluated. Further analysis of azurophilic granules during tracheal injury, and the impact of the release of their contents (elastase, cathepsin G) in NETs formation during induction of tracheal sterile injury and pain^64^, ^65^ also merit examination. We can only speculate that improving local epithelial protection with bias of neutrophils to a less injurious phenotype induced by CQ/NAC may be sufficient to prevent local bacterial contamination.

In summary, this study provides evidence that ETT contact with tracheal mucosa results in tissue damage partly induced by neutrophil migration and necrosis. Persistent local release of mtDNA promotes IL‐8 release in a TLR9/NF‐κB dependent manner. Interestingly, TLR9 has been shown to be prominent in autoimmune diseases; thus our view is that injury related to ETT is an iatrogenic induced autoimmune reaction. Our intervention with CQ/NAC was sufficient to ameliorate local neutrophil activation and tracheal damage. Future therapeutic interventions directed to mucosal activation of the mtDNA/TLR9/NF‐κB axis may help in prevention of mucosal damage, pain, and infection driven by medical devices.

## Supporting information


**Supplemental Figure 1**. Swine tracheal tissues stained with H&E for histologic evaluation of additional uncoated (A) with arrows identifying areas of tissue damage and loss of ciliary structures; and CQ/NAC‐coated (B) with arrows showing areas of tissue and cilia preservation, at 100X and 200X magnification.

**Supplemental Figure 2**. Corresponding FACS plots for Annexin V/7AAD staining for TLF cells from uncoated and CQ/NAC‐coated ETTs at hour 6. Live cells: 7‐AAD^−^ Annexin V^−^; necrotic cells: 7‐AAD^+^ Annexin V^−^; apoptotic cells: 7‐AAD^+^ Annexin V^+^; early apoptotic cells: 7‐AAD^−^ Annexin V^+^.

**Supplemental Figure 3**. Statistical analysis of cell viability in neutrophil cells obtained from TLF. Analysis conducted for live, early apoptotic, late apoptotic and necrotic cells following staining with 7AAD and Annexin V. Data represents 5 independent experiments per group where *P < 0.05 considered significant.

**Supplementary Table 1**. Normal Tracheal Tissue Histology. Histology of trachea not previously exposed to a foreign body (ETT). Representative histological images included in Figure 1A.
