# Supplementary figures and images for: Endotracheal intubation results in acute tracheal damage induced by mtDNA/TLR9/NF‐κB activity
Source: J Leukoc Biol. 2018 Dec 13;105(3):577–87. doi: 10.1002/JLB.5A0718-254RR (PMC7379990; doi:10.1002/JLB.5A0718-254RR)

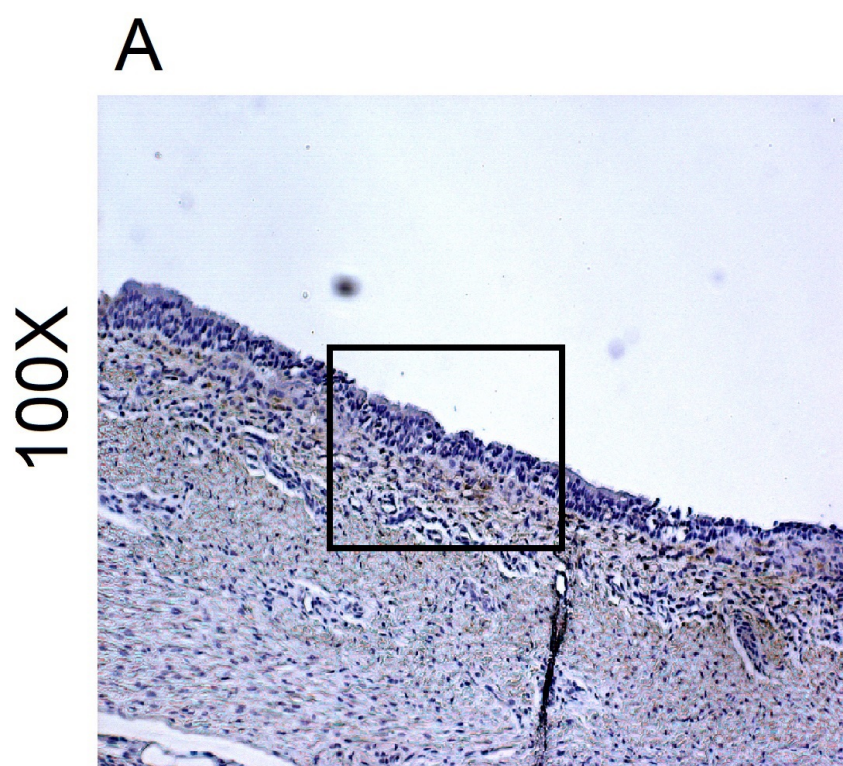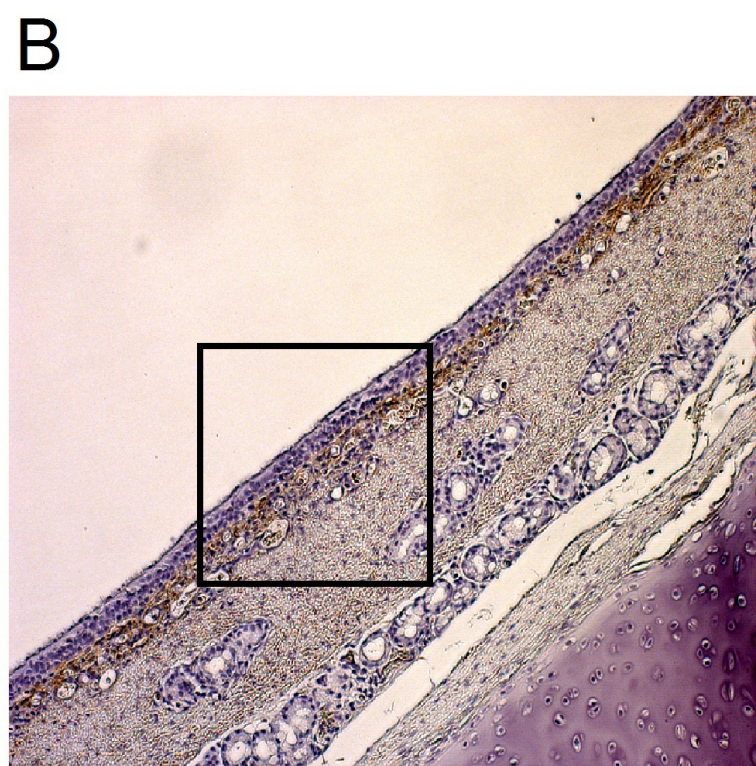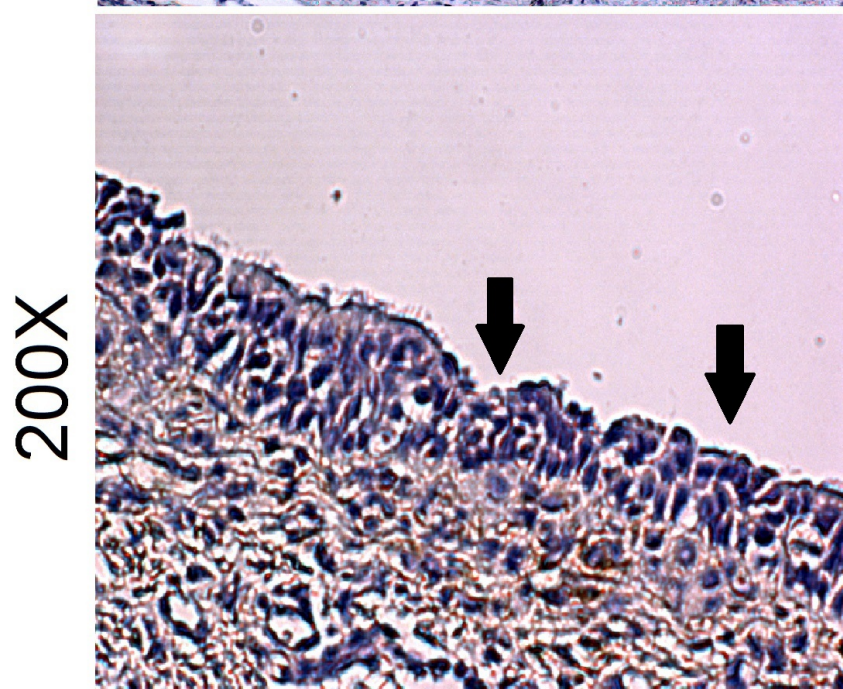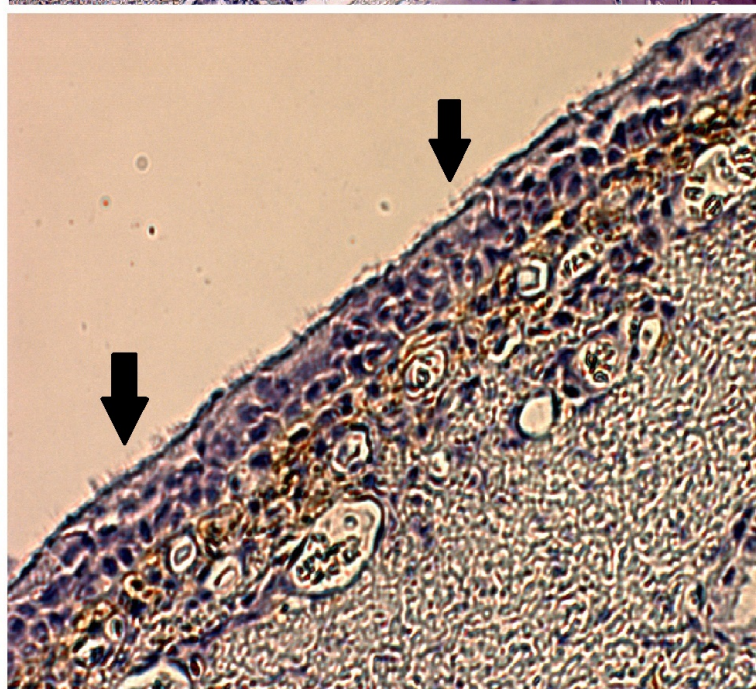

Supplement: Supplementary file 1 — Supplemental Figure 1. Swine tracheal tissues stained with H&E for histologic evaluation of additional uncoated (A) with arrows identifying areas of tissue damage and loss of ciliary structures; and CQ/NAC‐coated (B) with arrows showing areas of tissue and cilia preservation, at 100X and 200X magnification. [file JLB-105-577-s001.pdf]

# Uncoated 6 Hr

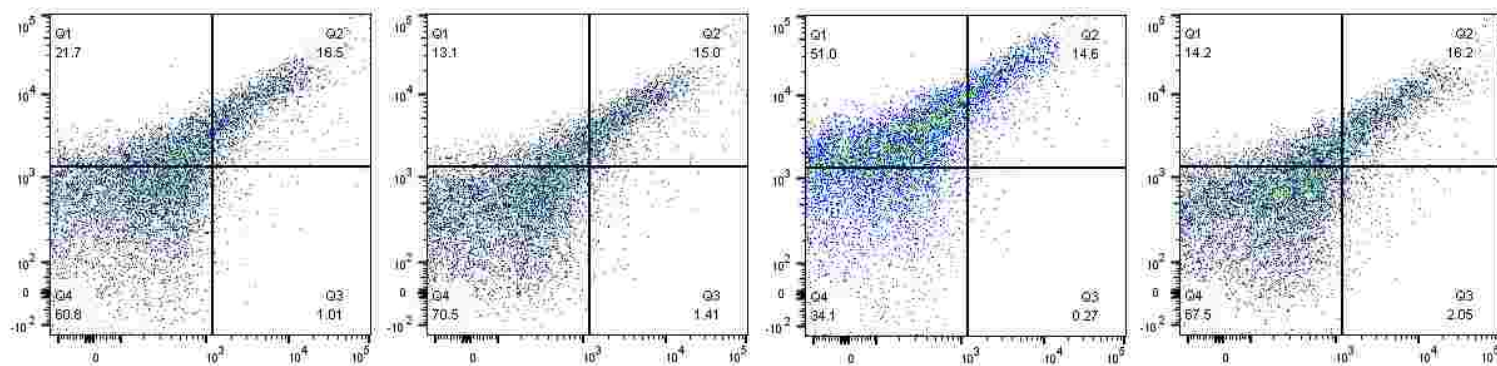

# CQ/NAC-Coated 6 Hr

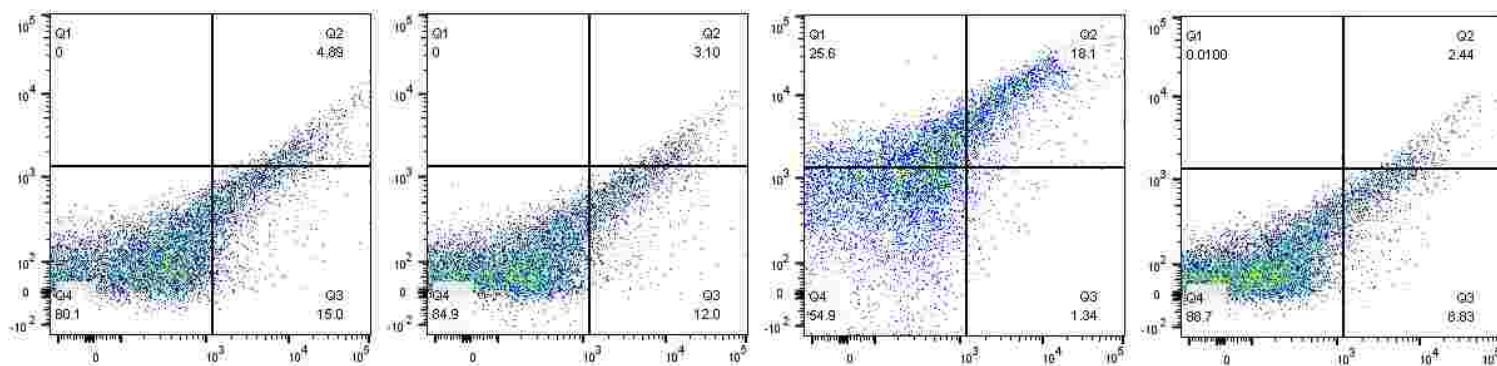

Supplement: Supplementary file 2 — Supplemental Figure 2. Corresponding FACS plots for Annexin V/7AAD staining for TLF cells from uncoated and CQ/NAC‐coated ETTs at hour 6. Live cells: 7‐AAD− Annexin V−; necrotic cells: 7‐AAD+ Annexin V−; apoptotic cells: 7‐AAD+ Annexin V+; early apoptotic cells: 7‐AAD− Annexin V+. [file JLB-105-577-s002.pdf]

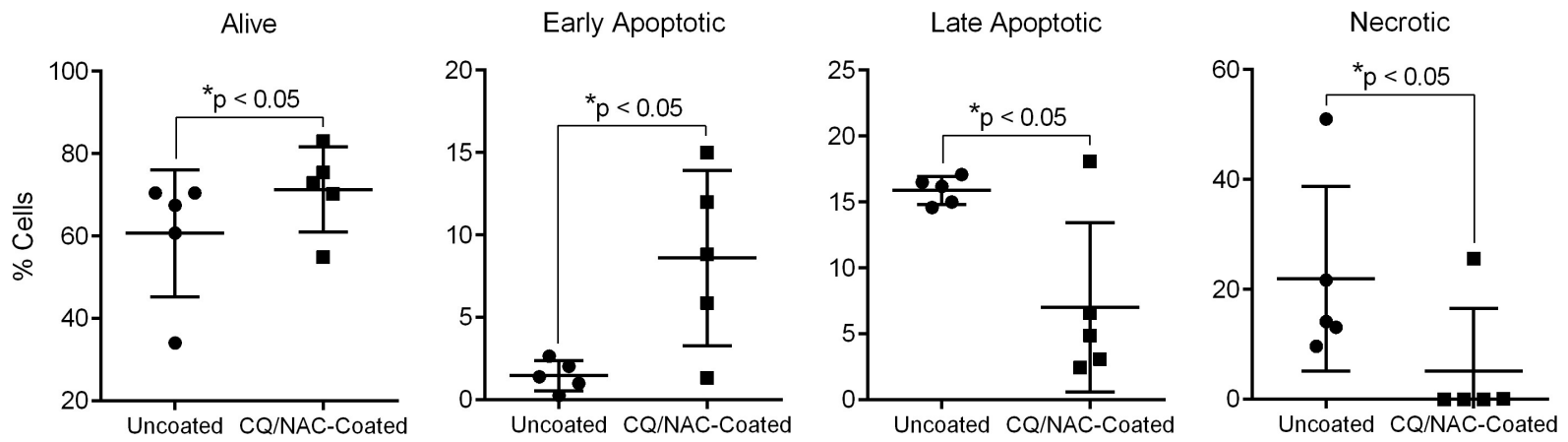

Supplement: Supplementary file 3 — Supplemental Figure 3. Statistical analysis of cell viability in neutrophil cells obtained from TLF. Analysis conducted for live, early apoptotic, late apoptotic and necrotic cells following staining with 7AAD and Annexin V. Data represents 5 independent experiments per group where *P < 0.05 considered significant. [file JLB-105-577-s003.pdf]
