# Supplementary material for: Endotracheal intubation results in acute tracheal damage induced by mtDNA/TLR9/NF‐κB activity
Source: J Leukoc Biol. 2018 Dec 13;105(3):577–87. doi: 10.1002/JLB.5A0718-254RR (PMC7379990; doi:10.1002/JLB.5A0718-254RR)
Supplement: Supplementary file 4 — Supplementary Table 1. Normal Tracheal Tissue Histology. Histology of trachea not previously exposed to a foreign body (ETT). Representative histological images included in Figure 1A. [file JLB-105-577-s004.pdf]

Variable	Subject 1	Subject 2	Subject 3	Subject 4
Criterion:				
Fraction of basement membrane covered by epithelium	0	1	1	3
Mucosal pathology	3	3	0	1
Submucosal pathology	0	0	0	0
Inflammation - Neutrophils	0.1	0.2	0	0
Inflammation - Mononuclear cells	2.8	2.2	2.8	2.6
Average Total Score	5.9	6.4	3.8	6.6

Supplementary Table 1. Normal Tracheal Tissue Histology. Histology of trachea not previously exposed to a foreign body (ETT). Representative histological images included in Figure 1A.
